# *DGUOK*-related mitochondrial DNA depletion syndrome presenting with neonatal cholestasis without marked hyperlactatemia: A diagnostic pitfall

**DOI:** 10.1016/j.ymgmr.2026.101314

**Published:** 2026-04-28

**Authors:** Moe Li, Hideo Sasai, Hiroaki Taniguchi, Atsushi Ishida, Yuya Kuwabara, Hideki Matsumoto, Daisuke Terazawa, Tomohiro Kanayama, Tatsuhiko Miyazaki, Akira Hara, Kei Murayama

**Affiliations:** a Department of Pediatrics, Gifu Prefectural Tajimi Hospital, 5-161 Maebata, Tajimi, Gifu 507-8522, Japan; b Department of Pediatrics and Neonatology, Nagoya City University Graduate School of Medical Sciences, 1 Kawasumi, Mizuho, Mizuho-ku, Nagoya, Aichi 467-0001, Japan; c Department of Early Diagnosis and Preventive Medicine for Rare Intractable Pediatric Diseases, Gifu University Graduate School of Medicine, 1-1 Yanagido, Gifu, Gifu 501-1194, Japan; d Department of Pediatrics, Gifu University Graduate School of Medicine, 1-1 Yanagido, Gifu, Gifu 501-1194, Japan; e Department of Tumor Pathology, Gifu University Graduate School of Medicine, 1-1 Yanagido, Gifu, Gifu 501-1194, Japan; f Department of Pathology, Gifu University Hospital, 1-1 Yanagido, Gifu, Gifu 501-1194, Japan; g Center for Medical Genetics, Department of Metabolism, Chiba Children's Hospital, 579-1 Heta, Midori-ku, Chiba, Chiba 266-0007, Japan; h Diagnostics and Therapeutics of Intractable Diseases, Intractable Disease Research Center, Graduate School of Medicine, Juntendo University, 3-1-3 Hongo, Bunkyo-ku, Tokyo 113-8431, Japan

**Keywords:** Cholestasis, Deoxyguanosine kinase deficiency, Hepatocerebral form, Liver transplantation, Mitochondrial diseases, Mitochondrial DNA depletion syndrome

## Abstract

Mitochondrial DNA depletion syndrome (MTDPS) is a group of severe mitochondrial disorders caused by nuclear gene variants that affect mitochondrial DNA (mtDNA) replication and nucleotide synthesis. Deoxyguanosine kinase deficiency is one of the most common subtypes, typically presenting with liver dysfunction in infancy and having a poor prognosis. We report a case of MTDPS presenting with cholestasis and mild hyperlactatemia in the neonatal period, which complicated early diagnosis. Histopathological and genetic analyses established the diagnosis. The patient, a female born at 36 weeks and 1 day of gestation, weighing 2124 g, developed cholestasis, poor feeding, and failure to thrive. Hyperlactatemia was not evident at presentation but gradually increased during the clinical course. Based on suspected mitochondrial disease, mitochondrial cocktail therapy was initiated on day 64. Liver transplantation was not feasible owing to cardiac and neurological complications, and conservative treatment was continued. However, the patient died of multiple organ failure on day 89. Postmortem liver biopsy showed a markedly reduced mtDNA copy number (8.1% of control), and genetic testing revealed a homozygous c.609_610del (p.Tyr204fs) variant in the *DGUOK* gene (NM_080916.3), confirming the diagnosis of *DGUOK*-related MTDPS. This case highlights that hyperlactatemia may be absent or only mild in the early stages of MTDPS, making timely diagnosis challenging. Mitochondrial functional analysis and genetic testing should be considered early in infants with unexplained cholestasis and liver failure, regardless of the lactate levels.

## Highlights

• Hyperlactatemia may be absent in some cases of early-stage MTDPS.
• Early mitochondrial analysis should be considered in unexplained cholestasis.
• Biopsy specimens should be frozen when the underlying hepatopathy is unknown.
• Liver transplantation is a treatment option for *DGUOK*-related MTDPS.
• Prompt diagnosis is essential to enable timely liver transplantation in MTDPS.

## 1 Introduction

Mitochondrial DNA depletion syndrome (MTDPS) is a group of severe mitochondrial disorders caused by nuclear gene variants that affect mitochondrial DNA (mtDNA) replication and nucleotide synthesis, resulting in multiple organ failure, particularly in the brain, liver, muscles, and kidneys. To date, more than 20 genes associated with MTDPS have been identified, including *DGUOK*, *MPV17*, and *POLG* [1]. *DGUOK* is among the most frequently implicated genes [2], located on chromosome 2p13 and encoding deoxyguanosine kinase (DGUOK), a key enzyme in mitochondrial nucleotide salvage pathways [3]. DGUOK deficiency is a rare disease, with an estimated prevalence of 1 in 110,000 births [4]. The initial manifestations typically include cholestasis, jaundice, hypotonia, feeding difficulties, and poor weight gain. Onset occurs during the neonatal period in 5.7% of cases and during infancy in 32.3%. As the disease progresses, patients develop metabolic acidosis, hypoglycemia, liver failure, and psychomotor developmental delay [2]. The prognosis is poor, with a median survival time of 0.5 years for neonatal-onset cases and 1.17 years for infantile-onset cases [2]. We report a neonatal case of *DGUOK*-related MTDPS presenting with cholestasis and mild hyperlactatemia, which may have contributed to delayed diagnosis.

## 2 Case presentation

**Fig. 1:**
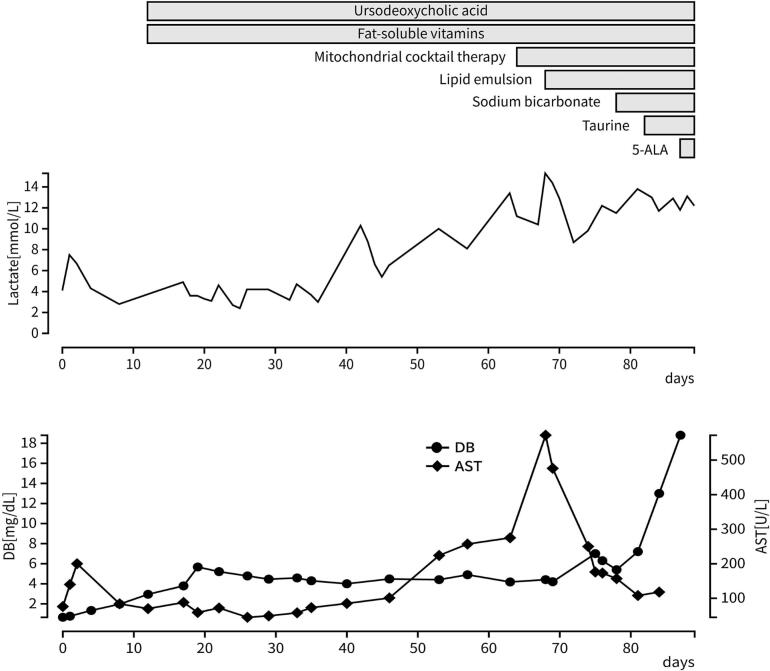
Clinical course of the patient. The time course of lactate, direct bilirubin (DB), and aspartate aminotransferase (AST) levels are shown. The duration and timing of medications (ursodeoxycholic acid, fat-soluble vitamins, mitochondrial cocktail therapy, lipid emulsion, sodium bicarbonate, taurine, and 5-aminolevulinic acid [5-ALA]) are indicated by horizontal bars.

The patient, a female infant, was born at 36 weeks and 1 day of gestation by elective cesarean section owing to a previous cesarean delivery. The mother was 37 years old, gravida 3, para 2, with no underlying diseases or complications during pregnancy. Both parents were of Filipino origin with no consanguinity. However, two maternal aunts had died in early childhood from an unexplained liver disease. At birth, the patient's height was 44.7 cm (−0.7 SD), weight was 2124 g (−1.0 SD), and head circumference was 31.8 cm (−0.2 SD). The Apgar score was 8 at 1 min and 9 at 5 min. The patient had no dysmorphic features, respiratory distress, or hepatosplenomegaly and muscle tone was normal. She was admitted for monitoring because of low birth weight. Although oral feeding was initiated on day 0, she developed hypoglycemia requiring intravenous glucose infusion until day 2. Owing to poor oral intake, tube feeding was introduced on day 6. At admission, blood tests revealed elevated gamma-glutamyltransferase (1858 U/L), although abdominal ultrasound showed no hepatobiliary abnormalities. On day 12, elevated direct bilirubin and total bile acids prompted treatment with ursodeoxycholic acid and fat-soluble vitamins. Enteral feeding was switched to medium-chain triglyceride (MCT) formula on day 15 because of poor weight gain. On day 17, grade II intraventricular hemorrhage according to the Papile classification was detected, and fresh frozen plasma was administered. Additional investigations for cholestasis were unremarkable (Table 1). Hepatobiliary scintigraphy on day 26 showed an absence of tracer excretion, suggestive of biliary atresia; however, laparoscopic cholangiography and liver biopsy on day 42 ruled out this diagnosis. Histopathological examination revealed hepatocellular cholestasis and pseudo-bile duct proliferation, without fibrosis or cirrhosis. Despite supportive therapy with MCT formula, ursodeoxycholic acid, and fat-soluble vitamins, lactate and liver transaminases progressively increased (Fig. 1). Biochemical tests on day 53 revealed elevated lactate/pyruvate ratio (45.8; normal range 7–20) and 3-hydroxybutyrate/acetoacetate ratio (7.9; normal range typically <2), which suggested mitochondrial disease. On day 64, mitochondrial cocktail therapy (thiamine, vitamin C, biotin, coenzyme Q10, and l-carnitine) was initiated. On day 68, the patient developed a fever with worsening lactic acidosis, and was transferred on day 69 to a tertiary care center. She was deemed ineligible for liver transplantation owing to impaired cardiac function and seizures suggestive of neurological involvement. On day 69, a skin biopsy was performed, and comprehensive genetic testing using next-generation sequencing-based targeted gene panel covering genes associated with inherited liver diseases and mitochondrial disorders was initiated, and adjunctive treatment with taurine and 5-aminolevulinic acid was started. Nevertheless, the patient died on day 89 owing to multiple organ failure.

**Table 1:** Laboratory data of the patient.

(day 12 of life)		(day 17 of life)			
AST (IU/L)	70	pH	7.3	FT4 (ng/dL)	1.4
ALT (IU/L)	37	pCO_2_ (mmHg)	52.1	TSH (mIU/L)	4.3
TB (mg/dL)	11.7	HCO_3_^−^ (mmol/L)	25.9	Ferritin (ng/mL)	323
DB (mg/dL)	3.0	BE (mmol/L)	−1.3	HBs-Ag	(−)
GGT (IU/L)	1292	AG (mmol/L)	8.3	HCV-Ab	(−)
LDH (IU/L)	471	Lactate (mmol/L)	4.1	CMV-IgM	(−)
Glucose (mg/dL)	60	PT-INR	1.6	CMV-IgG	(+)
Ammonia (μg/dL)	82	APTT (second)	56	CMV- PCR (urine)	(−)
TBA (μmol/L)	149.7	Fibrinogen (mg/dL)	63	HSV-PCR (blood)	(−)
		PIVKA-II (mAU/mL)	27		
(day 53 of life)					
L/P ratio: 45.8 (Lactate 10.2 mmol/L, Pyruvate 227 μmol/L),					
3OHB/AcAc ratio: 7.9 (3OHB 704 μmol/L, AcAc 89 μmol/L)					

**Amino acid analysis (day 12 of life)**					
(blood) Elevated: Tau, Hypro, Thr, Ser, Asn, Glu, Pro, α-ABA, Met, Cysthio, Leu, Tyr, His					
Ala/Lys ratio 2.40, Thr/Ser ratio 0.90, Cit/Ser ratio 0.14					
(urine) generalized aminoaciduria					

**Urinary organic acid analysis (day 19 of life)**					
marked increase in tyrosine metabolites, normal succinylacetone					
dicarboxylic aciduria, mild ketosis					

Abbreviations: AST, aspartate aminotransferase; ALT, alanine aminotransferase; TB, total bilirubin; DB, direct bilirubin; GGT, gamma- glutamyltransferase; LDH, lactate dehydrogenase; TBA, total bile acids; pCO2, partial pressure of carbon dioxide; HCO3-, bicarbonate; BE, base excess; AG, anion gap; PT-INR, prothrombin time-international normalized ratio; APTT, activated partial thromboplastin time; PIVKA-II, protein induced by vitamin K absence or antagonist-II; FT4, free thyroxine; TSH, thyroid-stimulating hormone; HBs—Ag, hepatitis B surface antigen; HCV-Ab, hepatitis C virus antibody; CMV, cytomegalovirus; IgM, immunoglobulin M antibody; IgG, immunoglobulin G antibody; PCR, polymerase chain reaction; HSV, herpes simplex virus; Pyruvate, pyruvic acid; L/P, lactate to pyruvate; 3OHB, 3-hydroxybutyrate; AcAc, acetoacetate; Tau, taurine; Hypro, hydroxyproline; Thr, threonine; Ser, serine; Asn, asparagine; Glu, glutamic acid; Pro, proline; α-ABA, α-amino-n-butyric acid; Met, methionine; Cysthio, cystathionine; Leu, leucine; Tyr, tyrosine; His, histidine; Ala, alanine; Lys, lysine; Cit, citrulline.

**Fig. 2:**
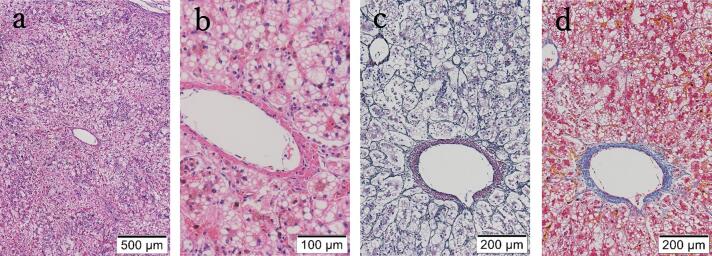
Liver histopathology of the patient. (a, b) Hepatocytes are markedly enlarged with anisocytosis, and exhibit both macrovesicular and microvesicular steatosis involving most hepatocytes (H&E staining). (c, d) No fibrosis or septal formation is observed in the portal areas (silver staining, Azan staining). The lobules demonstrate cholestasis with bile plugs.

Postmortem pathological examination revealed hepatocellular steatosis, ballooning, cholestasis, and bile duct proliferation, consistent with MTDPS (Fig. 2). Mitochondrial respiratory chain enzyme analyses showed decreased activities of complexes I, III, and IV with high activity of citrate synthase in liver tissue, whereas normal activities were observed in skin fibroblasts (Table 2). Quantification of mitochondrial DNA showed a marked reduction in the liver (8.1%) compared with the level in muscle (55.7%). Comprehensive genetic testing using next-generation sequencing identified a homozygous c.609_610del (p.Tyr204fs) variant in the *DGUOK* gene (NM_080916.3). The same variant was detected in a heterozygous state in both parents, confirming its parental origin. This variant has previously been reported [5] and is classified as pathogenic in ClinVar (Variation ID: 8156). Loss-of-function variants in *DGUOK* are known to disrupt the mitochondrial deoxynucleotide pool, leading to mtDNA depletion and/or multiple deletions [6]. Based on these findings, the patient was diagnosed with hepatocerebral MTDPS caused by a *DGUOK* variant.

**Table 2:** Mitochondrial respiratory chain enzyme activities of the patient.

%	Co I	Co II	Co III	Co IV	CS
Liver					
% of normal	6.5	58.4	10.1	31.1	310.8
CS ratio	2.0	18.0	3.2	9.6	–
Co II ratio	11.0	–	16.7	46.1	–
Fibroblast					
% of normal	70.5	47.5	143.6	68.9	97.5
CS ratio	71.4	48.3	142.2	69.7	–
Co II ratio	146.5	–	290.8	140.6	–

Co I, Complex I; Co II, Complex II; Co III, Complex III; Co IV, Complex IV; CS, citrate synthase.

## 3 Discussion

The case described in this paper is characterized by neonatal cholestasis, which is a relatively common manifestation of DGUOK deficiency. In one cohort study, cholestasis was reported in 57.5% of patients, making it the third most common symptom after elevated transaminases and jaundice [2]. However, neonatal cholestasis has a broad differential diagnosis. The most common cause is biliary atresia (25%–40%), followed by alpha-1 antitrypsin deficiency (10%–20%), Alagille syndrome (2%–14%), and progressive familial intrahepatic cholestasis (5%) [7]. Other etiologies include various infections, endocrine disorders, and metabolic disorders. Because biliary atresia is the most urgent diagnosis to exclude, it was prioritized in our patient and ruled out by laparoscopic cholangiography and liver biopsy. The histopathological findings were compatible with mitochondrial hepatopathy but were not diagnostic.

Although MTDPS is a rare cause of cholestasis, early recognition is essential because diagnosis requires specific tests and the disease is progressive with a poor prognosis. In the present case, hyperlactatemia was only mild (generally <4 mmol/L) in the neonatal period, which hindered early recognition. While MTDPS is known to present with hyperlactatemia, approximately 10% of cases do not exhibit hyperlactatemia at diagnosis [8]. In a cohort study of DGUOK deficiency, only 5% of cases showed lactic acidosis at onset, and 58.6% developed metabolic acidosis later [2]. In previous case reports, lactate levels ranged from 4.0 to 22.2 mmol/L (median 7.2 mmol/L) [9], [10], [11], [12]. The lactate levels can be influenced by hypoxia, hypoperfusion, or sampling conditions; therefore, interpretation requires caution. Laboratory findings suggestive of mitochondrial hepatopathy include the following: (1) persistent lactate levels ≥2.5 mmol/L, (2) lactate/pyruvate ratio ≥ 20, and (3) 3-hydroxybutyrate/acetoacetate ratio ≥ 2 [13]. These abnormalities reflect underlying mitochondrial dysfunction [14], which impairs oxidative phosphorylation, leading to reduced nicotinamide adenine dinucleotide (NAD^+^) regeneration and a shift toward anaerobic metabolism. Consequently, pyruvate is converted to lactate. In addition, an altered redox state promotes conversion of acetoacetate to 3-hydroxybutyrate. Therefore, when hyperlactatemia is persistent, mitochondrial hepatopathy should be considered, and further investigation is warranted. Multiple amino acids, including tyrosine, were elevated in the present case, consistent with previous reports of DGUOK deficiency [2]. However, these abnormalities are not specific and can be observed in various forms of liver dysfunction. In addition, hyperlactatemia may increase alanine levels; this was not observed in our patient, although the alanine/lysine ratio was mildly elevated. Mild hyperferritinemia also required differentiation from neonatal hemochromatosis, but has been reported in DGUOK deficiency [10]. Such findings highlight the difficulty of distinguishing mitochondrial hepatopathy from other metabolic disorders.

When mitochondrial hepatopathy is clinically suspected, mitochondrial functional analyses—such as mitochondrial respiratory chain enzyme analysis and mtDNA quantification—along with comprehensive genetic testing should be performed. In Japan, with recent advances in genetic diagnostic technologies, an increasing number of patients undergo early genetic testing using next-generation sequencing. However, genetic testing alone may be insufficient to establish a diagnosis in some patients. Therefore, when mitochondrial hepatopathy is included in the differential diagnosis, mitochondrial functional analyses continue to play an important role in establishing a definitive diagnosis. For liver analysis, the biopsy specimen must be non-formalin-fixed and frozen at −80 °C. In the present case, although a liver biopsy was performed on day 42, the specimen was formalin-fixed and not frozen. Therefore, respiratory chain enzyme analysis was not possible when MTDPS was later suspected. Freezing of biopsy specimens should be considered to facilitate the early diagnosis of MTDPS in infants with unexplained cholestasis.

To date, over 80 pathogenic variants of DGUOK deficiency have been identified. Clinical phenotypes are classified as hepatocerebral (58.8%), hepatopathic (21.9%), hepatomyocerebral (9.6%), and myopathic (9.6%) [2]. While the genotype–phenotype correlation remains unclear for many missense variants, frameshift variants such as that in the present case are associated with the neonatal or infantile hepatocerebral form [4]. Some patients without neurological symptoms may survive longer, but the hepatocerebral form typically presents in the neonatal period with median survival of only 0.42 years (range: <1 month to 17 years) [2]. The c.609_610del(p.Tyr204fs) variant identified in the present case was previously reported to be pathogenic. It was described in a patient with the hepatocerebral form, including jaundice, liver dysfunction, poor weight gain, and developmental delay, and the patient died at 6 months of age [5].

Currently, liver transplantation is the only treatment for liver failure in MTDPS. In mitochondrial hepatopathies overall, the survival rate after liver transplantation is approximately 30%, with poor outcomes due to neurological and extrahepatic symptoms [15]. In Japan, the survival rate after liver transplantation for hepatocerebral MTDPS is 41.7%, with fatal cases often being due to infections, pulmonary hypertension or heart failure [16]. In DGUOK deficiency, the median survival time is 1.92 years with transplantation (range: 1 month to 26 years), compared with 0.5 years without it [2]. These findings suggest that transplantation may prolong survival in selected patients; however, in previous reports, severe neurological symptoms have generally made the patients ineligible [17], [18]. Nystagmus, severe hypotonia, and marked developmental delay are known as predictors of poor post-transplant prognosis. For patients with mild neurological symptoms, outcomes are uncertain, and prognostic prediction using MRI or MRS remains difficult [2], [17]. Some transplanted patients have shown long-term neurological stability whereas others developed new or progressive symptoms. Therefore, the role of liver transplantation in DGUOK deficiency remains controversial, and decisions on whether to perform it in patients without neurological symptoms should be taken on a case-by-case basis [19]. In the present case, disease presentation occurred in the neonatal period and mortality followed at 0.24 years of age, consistent with the rapid course of DGUOK deficiency. It remains uncertain whether earlier liver transplantation prior to neurological involvement could have altered the clinical course.

In the present case, challenges in establishing a timely diagnosis may have been influenced by access to genetic testing and the availability of a clinical geneticist at the treating center. This highlights potential differences in care between centers with and without access to specialized genetic resources. Improving recognition of MTDPS among general pediatricians may facilitate earlier referral for appropriate diagnostic evaluation and timely diagnosis.

## 4 Conclusions

We report a neonatal case of *DGUOK*-related MTDPS presenting with cholestasis and only mild hyperlactatemia, which may have contributed to delayed diagnosis. This report emphasizes the need to consider MTDPS as a differential diagnosis in infants with unexplained cholestasis or liver failure, even in the absence of marked lactic acidosis. Clinicians should recognize that biochemical findings may be nonspecific and that appropriate preservation of biopsy specimens, including freezing to enable mitochondrial functional analysis, is crucial for timely diagnosis when MTDPS is suspected. Although the prognosis remains poor, early diagnosis and consideration of liver transplantation before neurological symptoms develop may improve outcomes in selected patients.

## Ethics statements

The original study to perform a genetic and functional analysis for mitochondrial diseases was approved by the Ethics Committee of Chiba Children's Hospital (approval no.: 2014-11-05), Juntendo University Graduate School of Medicine (approval no.: M17–0089-M01), and Gifu University Graduate School of Medicine (approval no.: 2023–261). The parents of the patient provided written informed consent for the publication of this report.

## CRediT authorship contribution statement

**Moe Li:** Investigation, Data curation, Writing – original draft. **Hideo Sasai:** Project administration, Data curation, Writing – review & editing. **Hiroaki Taniguchi:** Data curation, Writing – review & editing. **Atsushi Ishida:** Supervision, Conceptualization, Writing – review & editing. **Yuya Kuwabara:** Data curation, Writing – review & editing. **Hideki Matsumoto:** Data curation, Writing – review & editing. **Daisuke Terazawa:** Data curation, Writing – review & editing. **Tomohiro Kanayama:** Investigation, Data curation, Writing – review & editing. **Tatsuhiko Miyazaki:** Investigation, Data curation, Writing – review & editing. **Akira Hara:** Supervision, Investigation, Data curation, Writing – review & editing. **Kei Murayama:** Supervision, Resources, Methodology, Investigation, Funding acquisition, Writing – review & editing.

## Funding

This work was supported by a grant for the Practical Research Project for Rare/Intractable Diseases from AMED (Fund ID: JP23ek0109625 and JP24lk0221189s0301) and 10.13039/501100003478Grants-in-Aid of the Research on Intractable Diseases from the Ministry of Health, Labour and Welfare of Japan (Grant Number 23FC1034 and 24FC1010) to KM.

## Declaration of competing interest

H.S. has received an endowed chair funded by Gifu Research Center for Public Health.

M.L., H.T., A.I., Y.K., H.M., D.T., T.K., T.M., A.H. and K.M. declare no conflicts of interest.

## Data Availability

Data will be made available on request.
